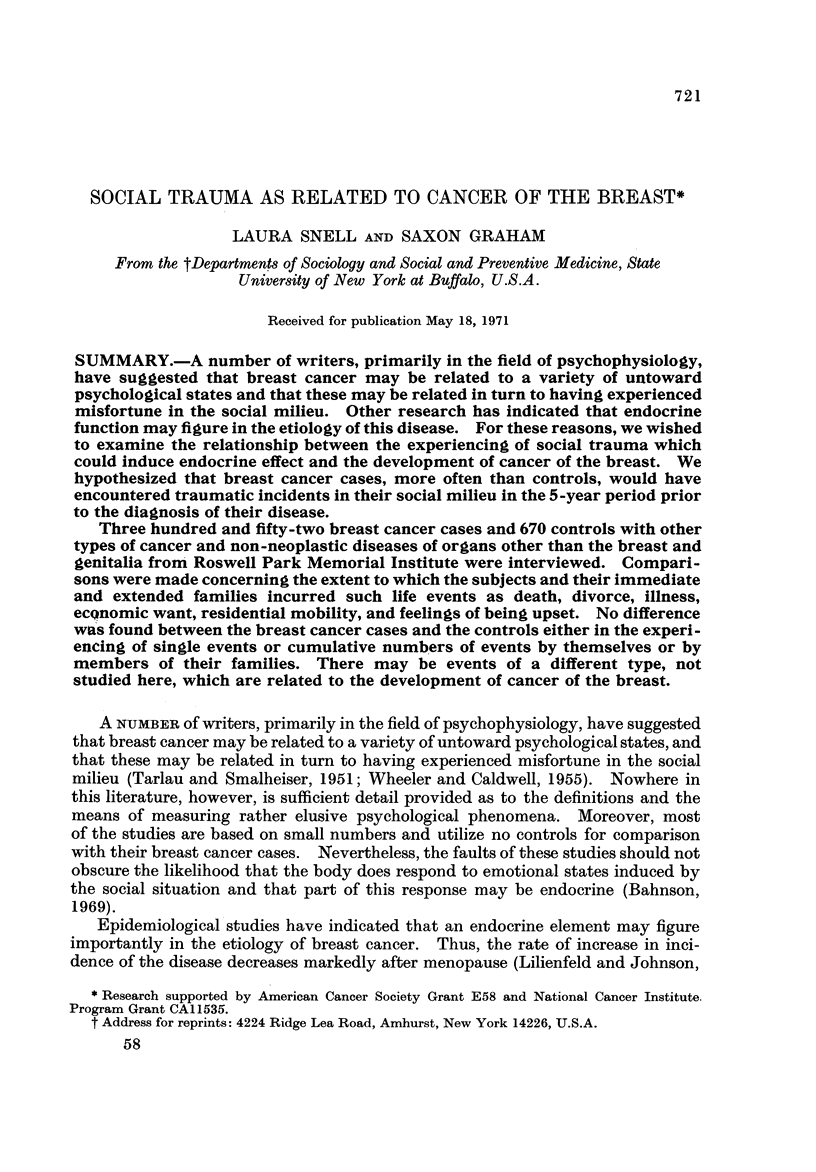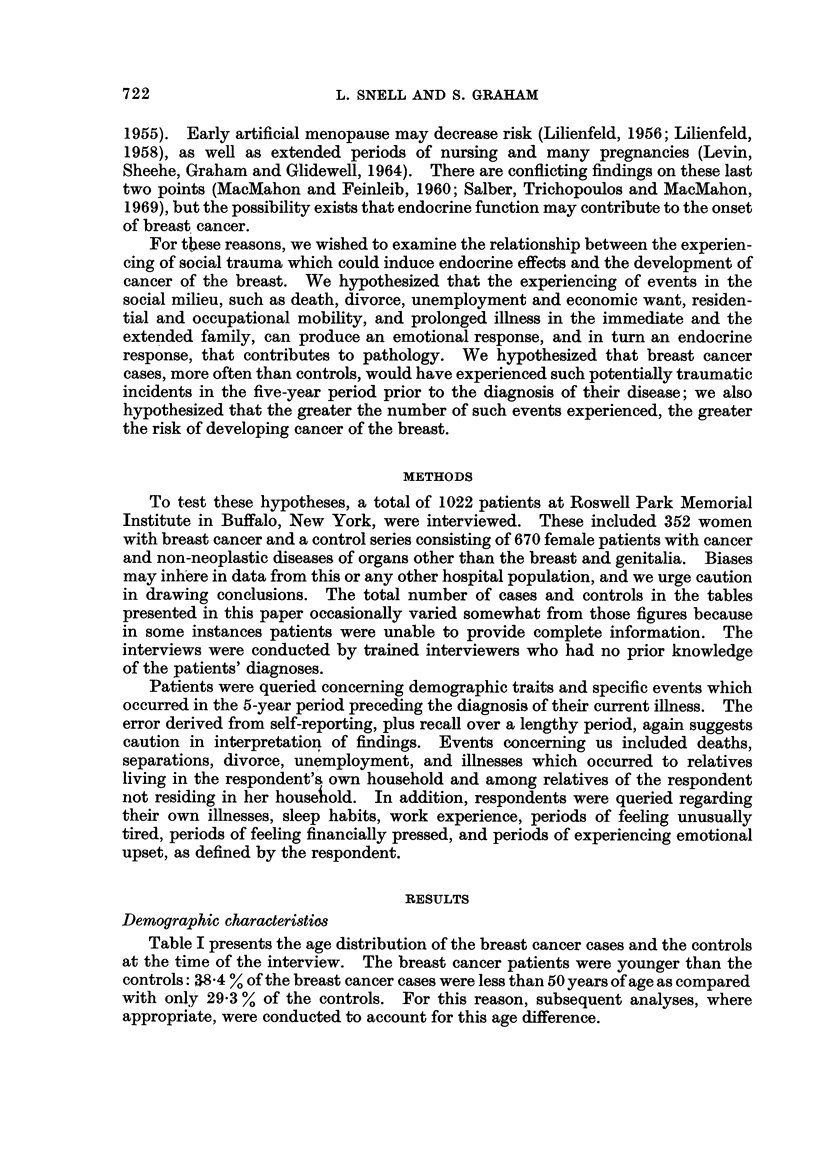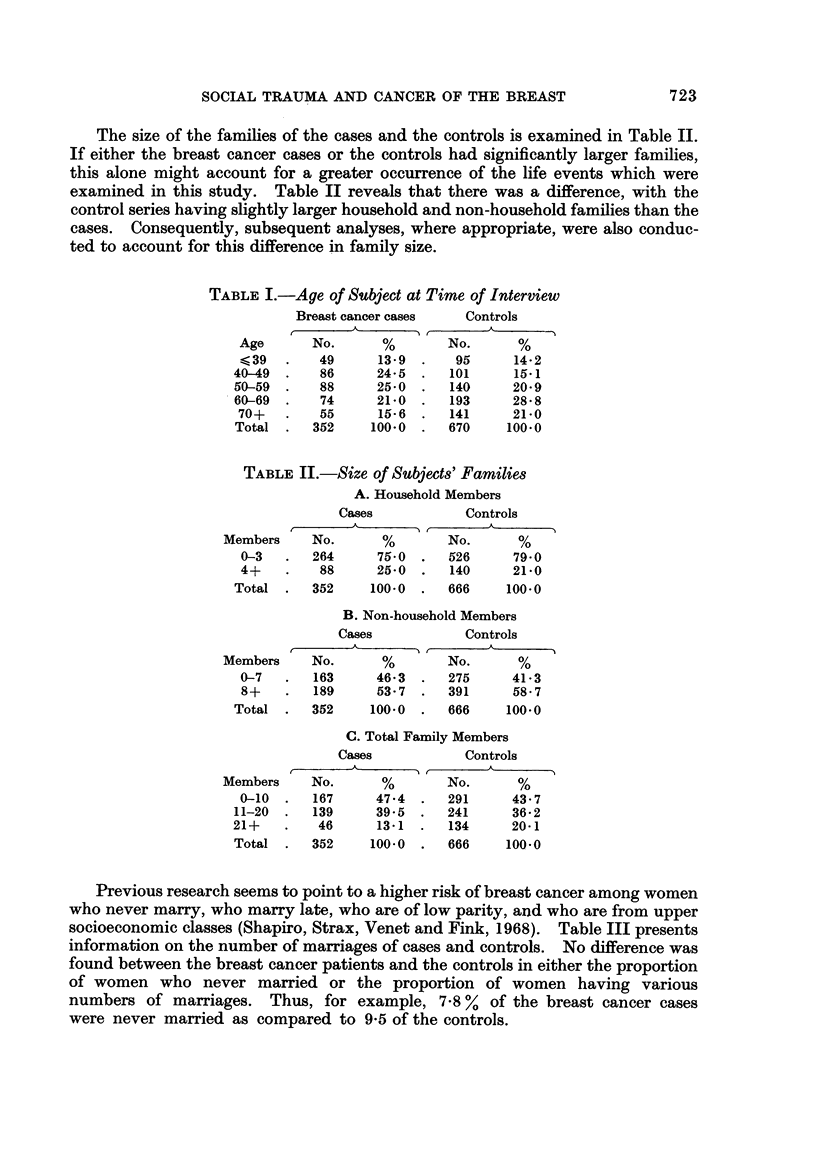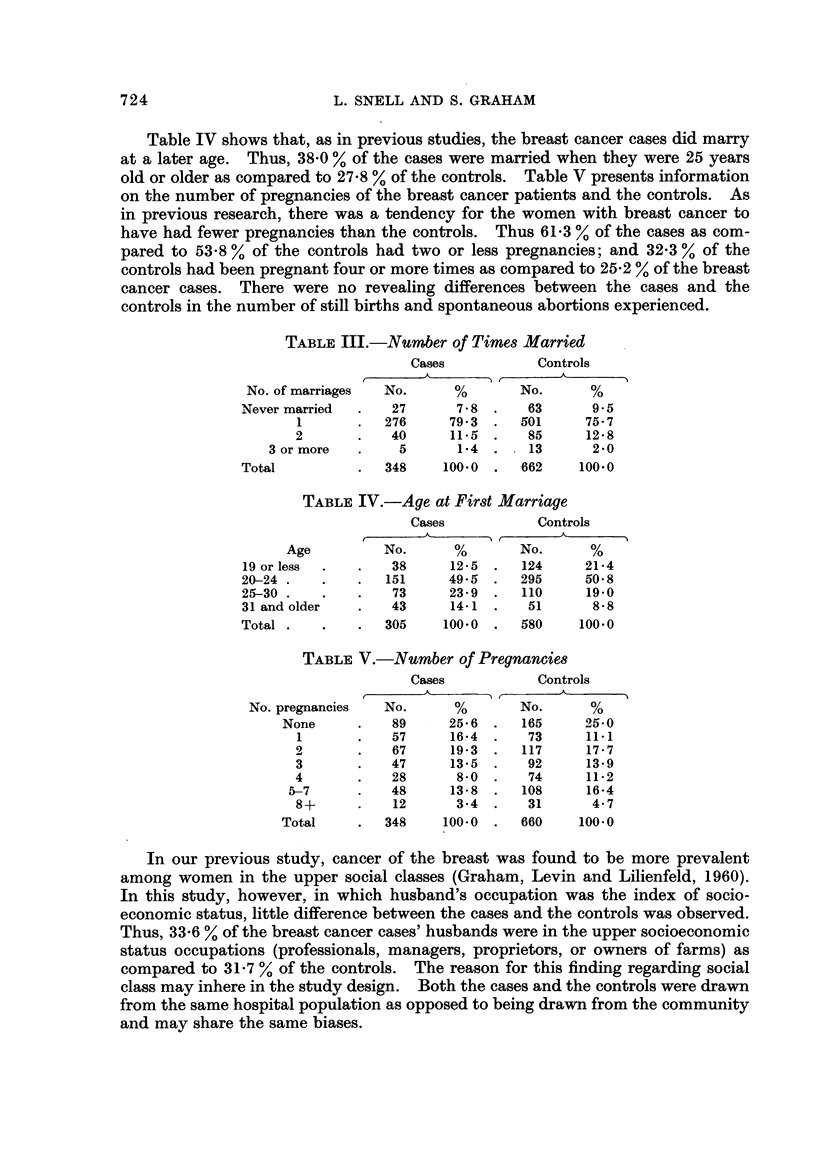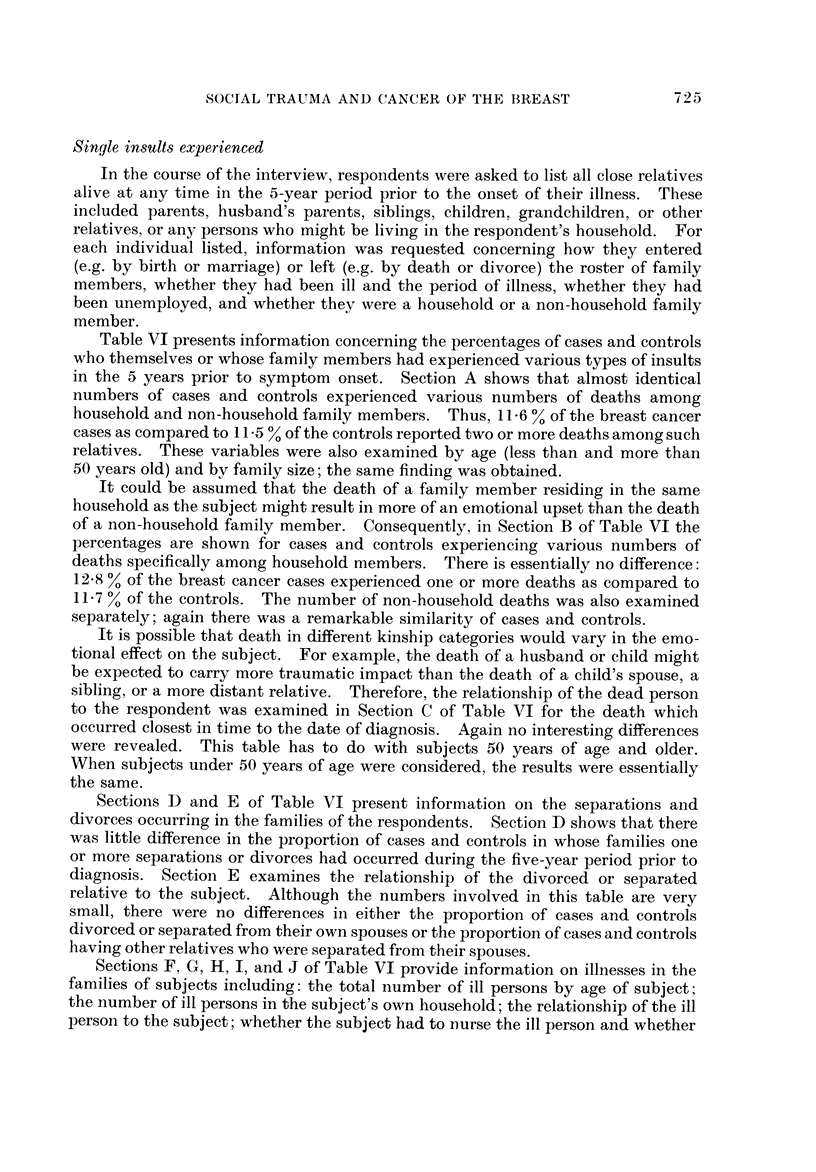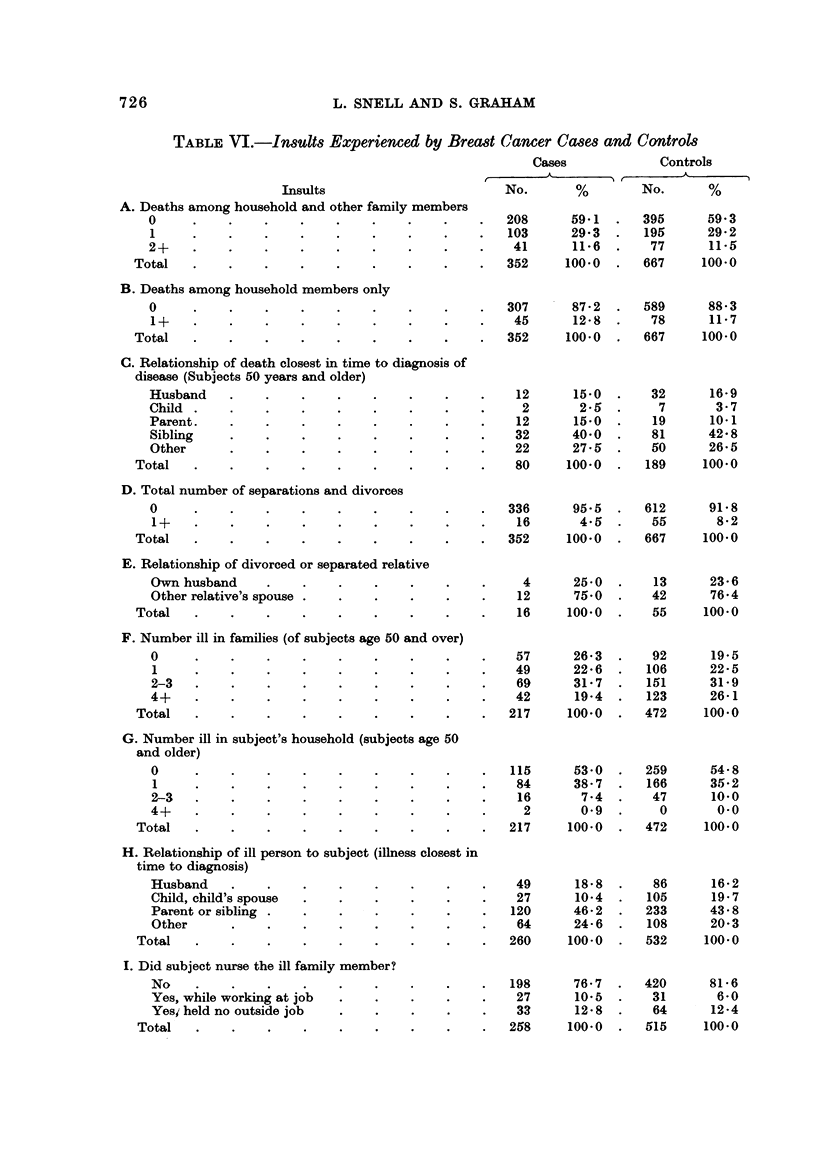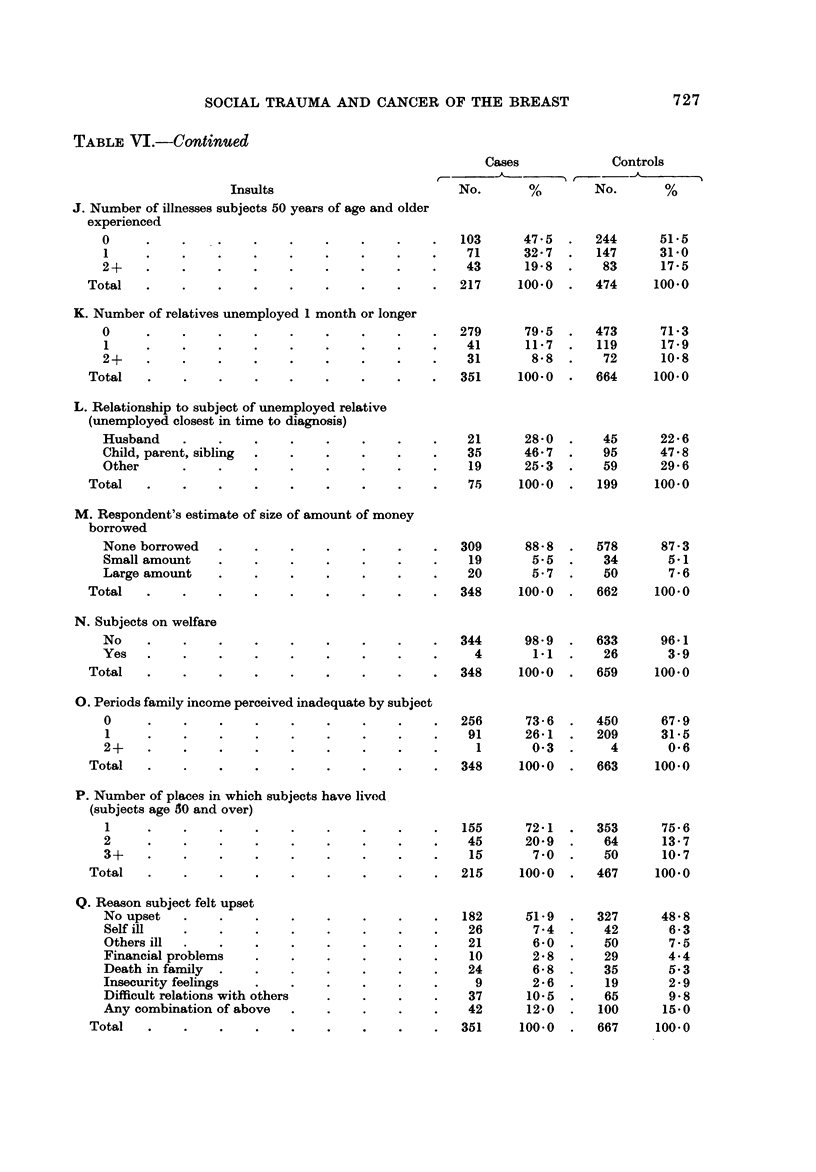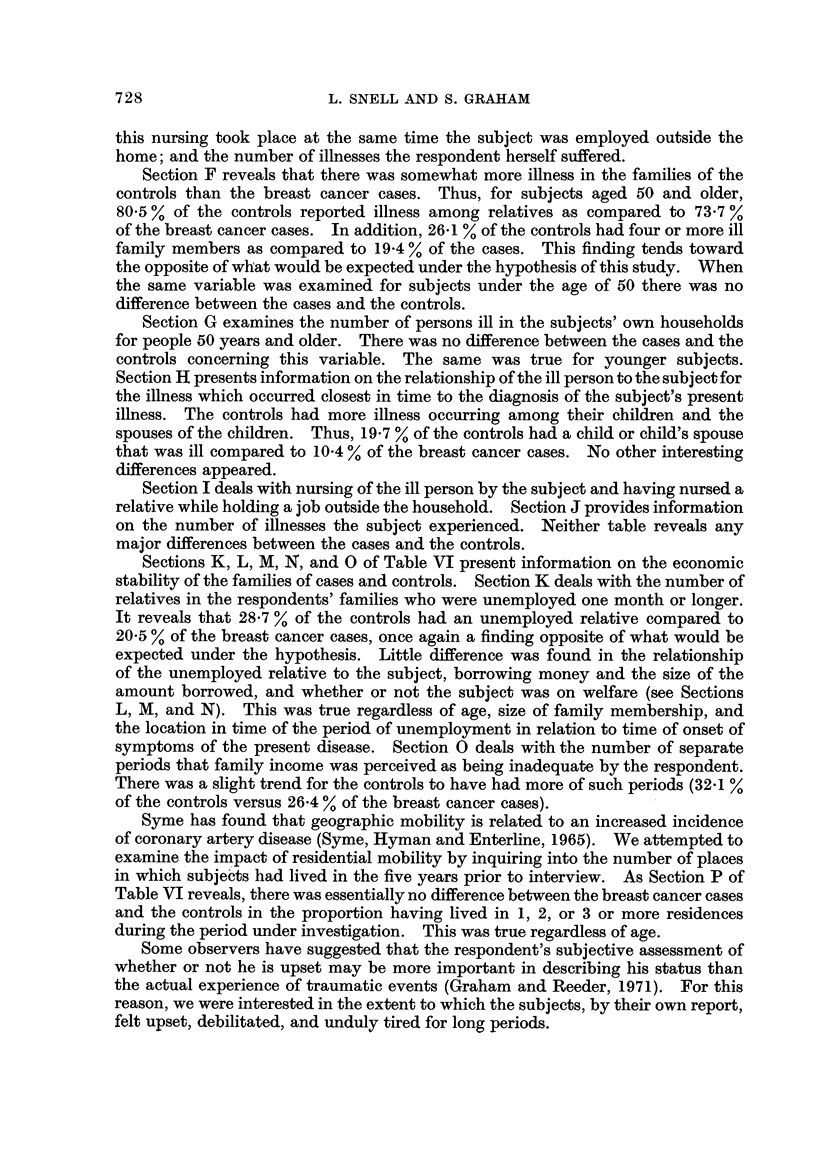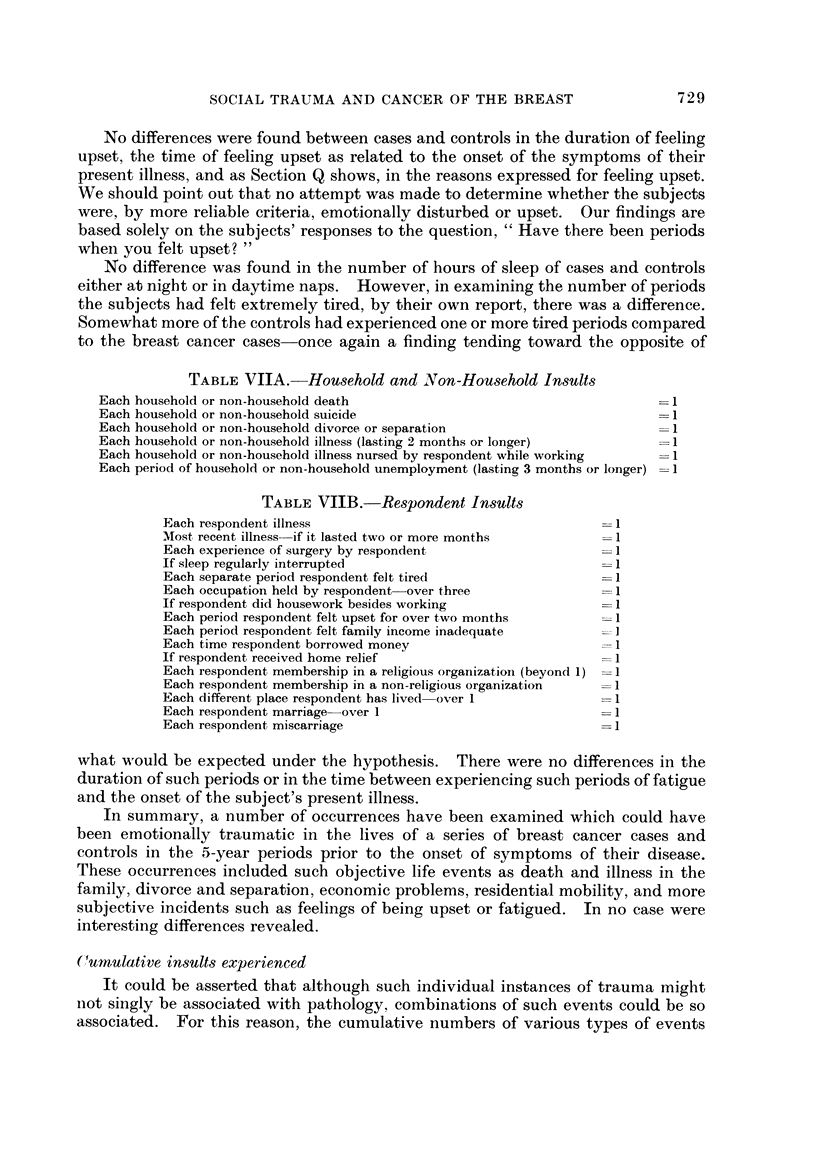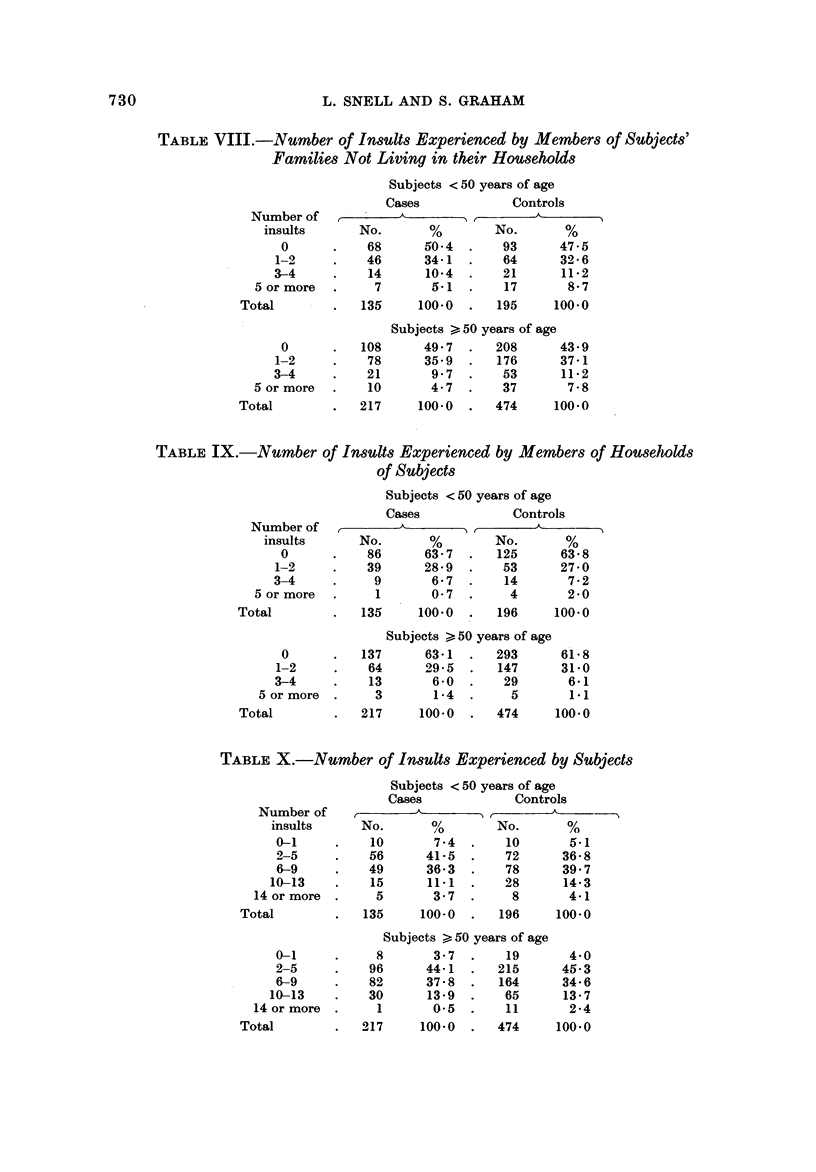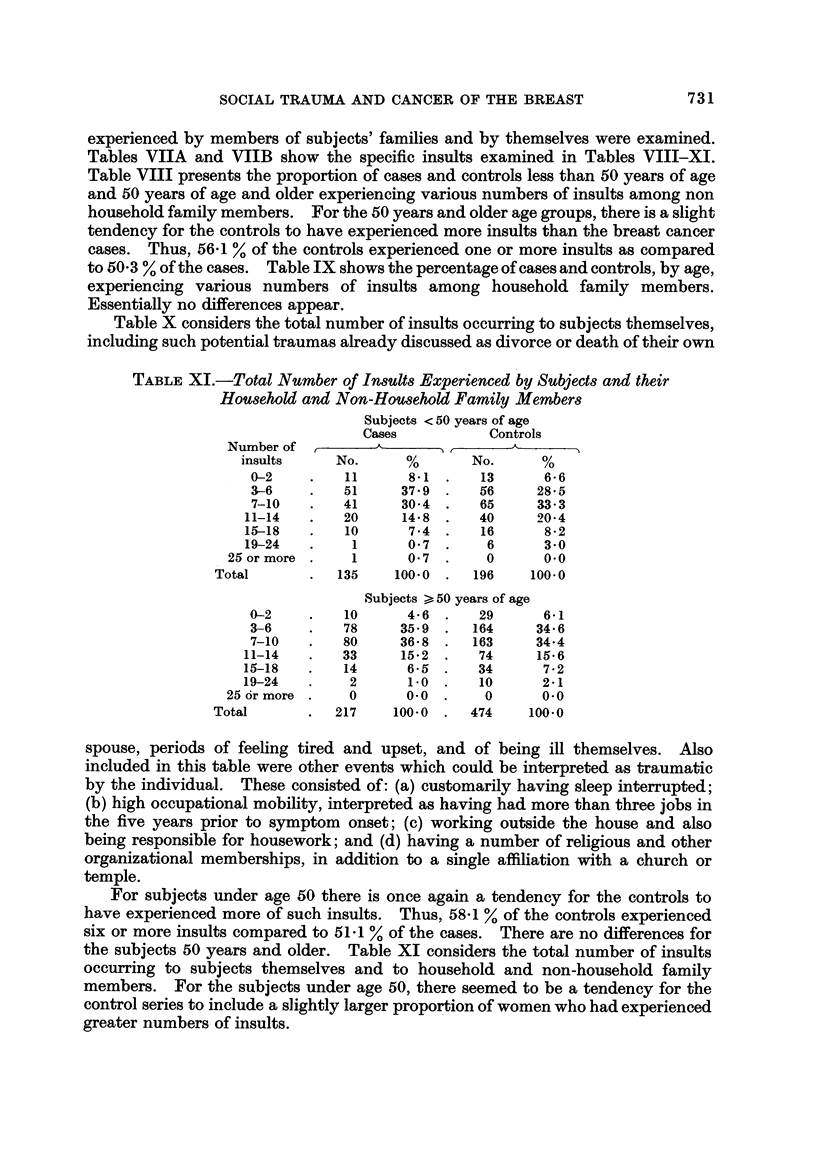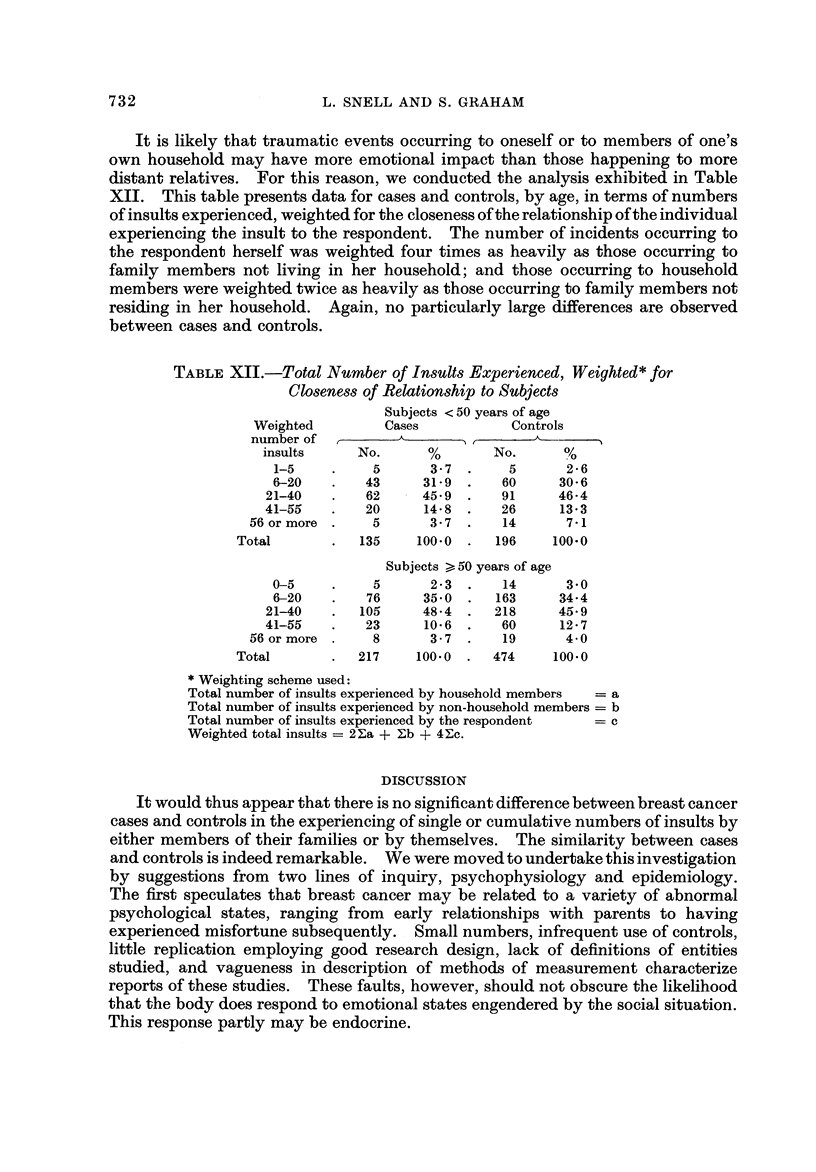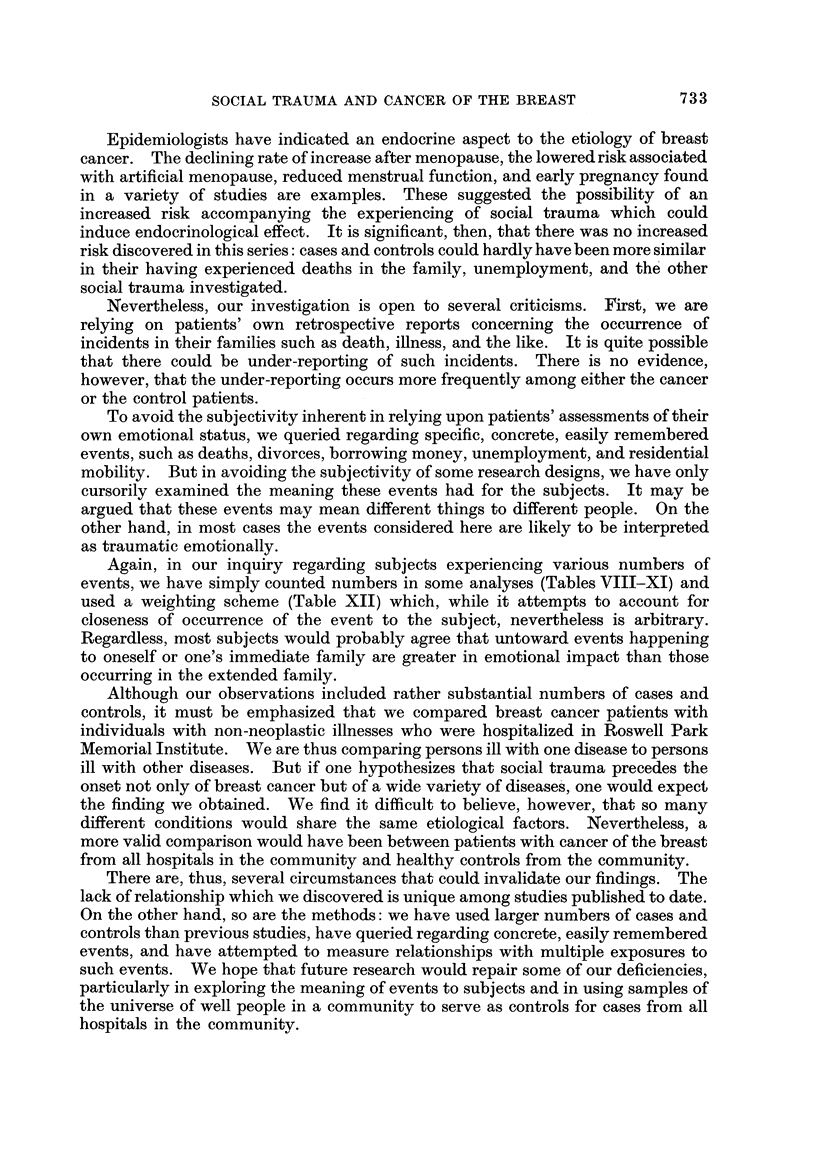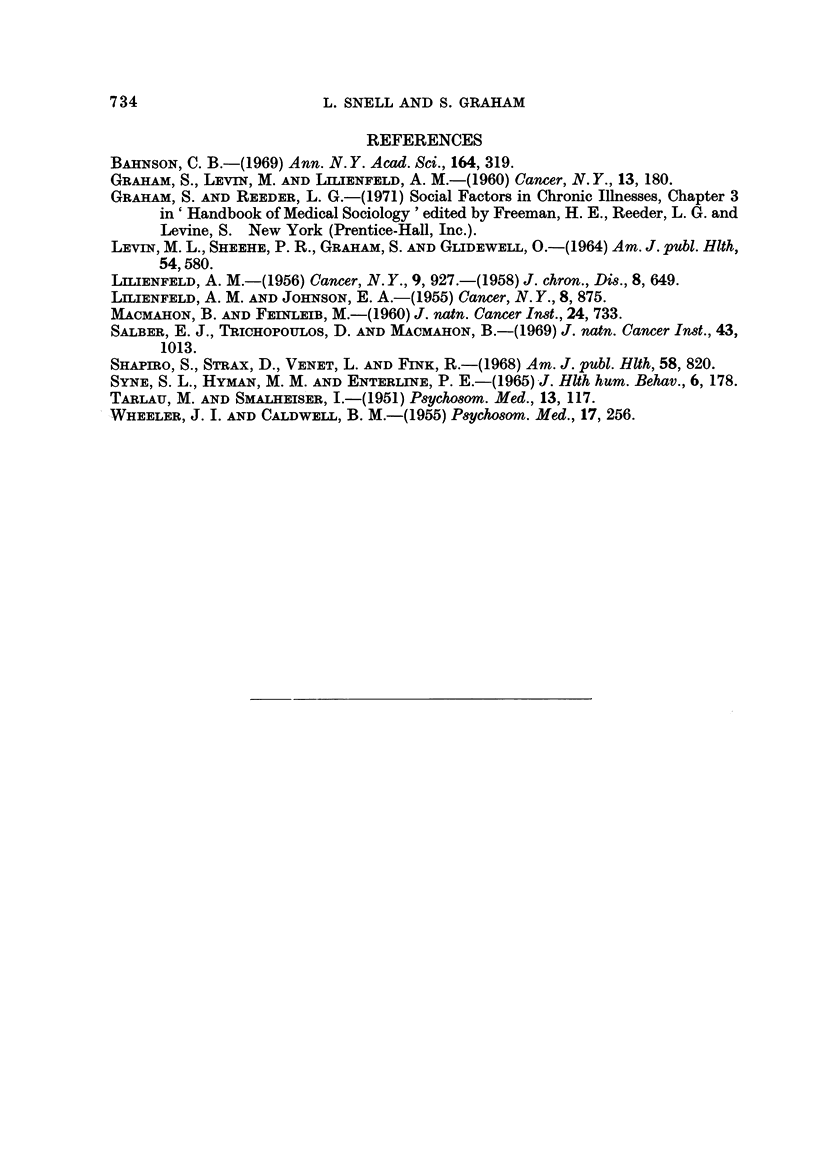# Social Trauma as Related to Cancer of the Breast

**DOI:** 10.1038/bjc.1971.87

**Published:** 1971-12

**Authors:** Laura Snell, Saxon Graham

## Abstract

A number of writers, primarily in the field of psychophysiology, have suggested that breast cancer may be related to a variety of untoward psychological states and that these may be related in turn to having experienced misfortune in the social milieu. Other research has indicated that endocrine function may figure in the etiology of this disease. For these reasons, we wished to examine the relationship between the experiencing of social trauma which could induce endocrine effect and the development of cancer of the breast. We hypothesized that breast cancer cases, more often than controls, would have encountered traumatic incidents in their social milieu in the 5-year period prior to the diagnosis of their disease.

Three hundred and fifty-two breast cancer cases and 670 controls with other types of cancer and non-neoplastic diseases of organs other than the breast and genitalia from Roswell Park Memorial Institute were interviewed. Comparisons were made concerning the extent to which the subjects and their immediate and extended families incurred such life events as death, divorce, illness, economic want, residential mobility, and feelings of being upset. No difference was found between the breast cancer cases and the controls either in the experiencing of single events or cumulative numbers of events by themselves or by members of their families. There may be events of a different type, not studied here, which are related to the development of cancer of the breast.


### SOCIAL TRAUMA AS RELATED TO CANCER OF THE BREAST*

LAURA SNELL AND SAXON GRAHAM

*From the †Departments of Sociology and Social and Preventive Medicine, State University of New York at Buffalo, U.S.A.*

Received for publication May 18, 1971

**SUMMARY.—A number of writers, primarily in the field of psychophysiology, have suggested that breast cancer may be related to a variety of untoward psychological states and that these may be related in turn to having experienced misfortune in the social milieu. Other research has indicated that endocrine function may figure in the etiology of this disease. For these reasons, we wished to examine the relationship between the experiencing of social trauma which could induce endocrine effect and the development of cancer of the breast. We hypothesized that breast cancer cases, more often than controls, would have encountered traumatic incidents in their social milieu in the 5-year period prior to the diagnosis of their disease.**

**Three hundred and fifty-two breast cancer cases and 670 controls with other types of cancer and non-neoplastic diseases of organs other than the breast and genitalia from Roswell Park Memorial Institute were interviewed. Comparisons were made concerning the extent to which the subjects and their immediate and extended families incurred such life events as death, divorce, illness, economic want, residential mobility, and feelings of being upset. No difference was found between the breast cancer cases and the controls either in the experiencing of single events or cumulative numbers of events by themselves or by members of their families. There may be events of a different type, not studied here, which are related to the development of cancer of the breast.**

A NUMBER of writers, primarily in the field of psychophysiology, have suggested that breast cancer may be related to a variety of untoward psychological states, and that these may be related in turn to having experienced misfortune in the social milieu (Tarlau and Smalheiser, 1951; Wheeler and Caldwell, 1955). Nowhere in this literature, however, is sufficient detail provided as to the definitions and the means of measuring rather elusive psychological phenomena. Moreover, most of the studies are based on small numbers and utilize no controls for comparison with their breast cancer cases. Nevertheless, the faults of these studies should not obscure the likelihood that the body does respond to emotional states induced by the social situation and that part of this response may be endocrine (Bahnson, 1969).

Epidemiological studies have indicated that an endocrine element may figure importantly in the etiology of breast cancer. Thus, the rate of increase in incidence of the disease decreases markedly after menopause (Lilienfeld and Johnson,

* Research supported by American Cancer Society Grant E58 and National Cancer Institute. Program Grant CA11535.

† Address for reprints: 4224 Ridge Lea Road, Amhurst, New York 14226, U.S.A.

1955). Early artificial menopause may decrease risk (Lilienfeld, 1956; Lilienfeld, 1958), as well as extended periods of nursing and many pregnancies (Levin, Sheehe, Graham and Glidewell, 1964). There are conflicting findings on these last two points (MacMahon and Feinleib, 1960; Salber, Trichopoulos and MacMahon, 1969), but the possibility exists that endocrine function may contribute to the onset of breast cancer.

For these reasons, we wished to examine the relationship between the experiencing of social trauma which could induce endocrine effects and the development of cancer of the breast. We hypothesized that the experiencing of events in the social milieu, such as death, divorce, unemployment and economic want, residential and occupational mobility, and prolonged illness in the immediate and the extended family, can produce an emotional response, and in turn an endocrine response, that contributes to pathology. We hypothesized that breast cancer cases, more often than controls, would have experienced such potentially traumatic incidents in the five-year period prior to the diagnosis of their disease; we also hypothesized that the greater the number of such events experienced, the greater the risk of developing cancer of the breast.

### METHODS

To test these hypotheses, a total of 1022 patients at Roswell Park Memorial Institute in Buffalo, New York, were interviewed. These included 352 women with breast cancer and a control series consisting of 670 female patients with cancer and non-neoplastic diseases of organs other than the breast and genitalia. Biases may inhere in data from this or any other hospital population, and we urge caution in drawing conclusions. The total number of cases and controls in the tables presented in this paper occasionally varied somewhat from those figures because in some instances patients were unable to provide complete information. The interviews were conducted by trained interviewers who had no prior knowledge of the patients' diagnoses.

Patients were queried concerning demographic traits and specific events which occurred in the 5-year period preceding the diagnosis of their current illness. The error derived from self-reporting, plus recall over a lengthy period, again suggests caution in interpretation of findings. Events concerning us included deaths, separations, divorce, unemployment, and illnesses which occurred to relatives living in the respondent's own household and among relatives of the respondent not residing in her household. In addition, respondents were queried regarding their own illnesses, sleep habits, work experience, periods of feeling unusually tired, periods of feeling financially pressed, and periods of experiencing emotional upset, as defined by the respondent.

### RESULTS

### *Demographic characteristics*

Table I presents the age distribution of the breast cancer cases and the controls at the time of the interview. The breast cancer patients were younger than the controls: 38·4 % of the breast cancer cases were less than 50 years of age as compared with only 29·3 % of the controls. For this reason, subsequent analyses, where appropriate, were conducted to account for this age difference.

The size of the families of the cases and the controls is examined in Table II. If either the breast cancer cases or the controls had significantly larger families, this alone might account for a greater occurrence of the life events which were examined in this study. Table II reveals that there was a difference, with the control series having slightly larger household and non-household families than the cases. Consequently, subsequent analyses, where appropriate, were also conducted to account for this difference in family size.

TABLE I.—*Age of Subject at Time of Interview*

| | Breast cancer cases | | Controls | |
|---|---|---|---|---|
| Age | No. | % | No. | % |
| ≤39 | 49 | 13·9 | 95 | 14·2 |
| 40–49 | 86 | 24·5 | 101 | 15·1 |
| 50–59 | 88 | 25·0 | 140 | 20·9 |
| 60–69 | 74 | 21·0 | 193 | 28·8 |
| 70+ | 55 | 15·6 | 141 | 21·0 |
| Total | 352 | 100·0 | 670 | 100·0 |

TABLE II.—*Size of Subjects' Families*

A. Household Members

| | Cases | | Controls | |
|---|---|---|---|---|
| Members | No. | % | No. | % |
| 0–3 | 264 | 75·0 | 526 | 79·0 |
| 4+ | 88 | 25·0 | 140 | 21·0 |
| Total | 352 | 100·0 | 666 | 100·0 |

B. Non-household Members

| | Cases | | Controls | |
|---|---|---|---|---|
| Members | No. | % | No. | % |
| 0–7 | 163 | 46·3 | 275 | 41·3 |
| 8+ | 189 | 53·7 | 391 | 58·7 |
| Total | 352 | 100·0 | 666 | 100·0 |

C. Total Family Members

| | Cases | | Controls | |
|---|---|---|---|---|
| Members | No. | % | No. | % |
| 0–10 | 167 | 47·4 | 291 | 43·7 |
| 11–20 | 139 | 39·5 | 241 | 36·2 |
| 21+ | 46 | 13·1 | 134 | 20·1 |
| Total | 352 | 100·0 | 666 | 100·0 |

Previous research seems to point to a higher risk of breast cancer among women who never marry, who marry late, who are of low parity, and who are from upper socioeconomic classes (Shapiro, Strax, Venet and Fink, 1968). Table III presents information on the number of marriages of cases and controls. No difference was found between the breast cancer patients and the controls in either the proportion of women who never married or the proportion of women having various numbers of marriages. Thus, for example, 7·8% of the breast cancer cases were never married as compared to 9·5 of the controls.

Table IV shows that, as in previous studies, the breast cancer cases did marry at a later age. Thus, 38·0 % of the cases were married when they were 25 years old or older as compared to 27·8 % of the controls. Table V presents information on the number of pregnancies of the breast cancer patients and the controls. As in previous research, there was a tendency for the women with breast cancer to have had fewer pregnancies than the controls. Thus 61·3 % of the cases as compared to 53·8 % of the controls had two or less pregnancies; and 32·3 % of the controls had been pregnant four or more times as compared to 25·2 % of the breast cancer cases. There were no revealing differences between the cases and the controls in the number of still births and spontaneous abortions experienced.

TABLE III.—*Number of Times Married*

| | Cases | | Controls | |
|---|---|---|---|---|
| No. of marriages | No. | % | No. | % |
| Never married | 27 | 7·8 | 63 | 9·5 |
| 1 | 276 | 79·3 | 501 | 75·7 |
| 2 | 40 | 11·5 | 85 | 12·8 |
| 3 or more | 5 | 1·4 | 13 | 2·0 |
| Total | 348 | 100·0 | 662 | 100·0 |

TABLE IV.—*Age at First Marriage*

| | Cases | | Controls | |
|---|---|---|---|---|
| Age | No. | % | No. | % |
| 19 or less | 38 | 12·5 | 124 | 21·4 |
| 20–24 | 151 | 49·5 | 295 | 50·8 |
| 25–30 | 73 | 23·9 | 110 | 19·0 |
| 31 and older | 43 | 14·1 | 51 | 8·8 |
| Total | 305 | 100·0 | 580 | 100·0 |

TABLE V.—*Number of Pregnancies*

| | Cases | | Controls | |
|---|---|---|---|---|
| No. pregnancies | No. | % | No. | % |
| None | 89 | 25·6 | 165 | 25·0 |
| 1 | 57 | 16·4 | 73 | 11·1 |
| 2 | 67 | 19·3 | 117 | 17·7 |
| 3 | 47 | 13·5 | 92 | 13·9 |
| 4 | 28 | 8·0 | 74 | 11·2 |
| 5–7 | 48 | 13·8 | 108 | 16·4 |
| 8+ | 12 | 3·4 | 31 | 4·7 |
| Total | 348 | 100·0 | 660 | 100·0 |

In our previous study, cancer of the breast was found to be more prevalent among women in the upper social classes (Graham, Levin and Lilienfeld, 1960). In this study, however, in which husband's occupation was the index of socioeconomic status, little difference between the cases and the controls was observed. Thus, 33·6 % of the breast cancer cases' husbands were in the upper socioeconomic status occupations (professionals, managers, proprietors, or owners of farms) as compared to 31·7 % of the controls. The reason for this finding regarding social class may inhere in the study design. Both the cases and the controls were drawn from the same hospital population as opposed to being drawn from the community and may share the same biases.

*Single insults experienced*

In the course of the interview, respondents were asked to list all close relatives alive at any time in the 5-year period prior to the onset of their illness. These included parents, husband's parents, siblings, children, grandchildren, or other relatives, or any persons who might be living in the respondent's household. For each individual listed, information was requested concerning how they entered (e.g. by birth or marriage) or left (e.g. by death or divorce) the roster of family members, whether they had been ill and the period of illness, whether they had been unemployed, and whether they were a household or a non-household family member.

Table VI presents information concerning the percentages of cases and controls who themselves or whose family members had experienced various types of insults in the 5 years prior to symptom onset. Section A shows that almost identical numbers of cases and controls experienced various numbers of deaths among household and non-household family members. Thus, 11·6 % of the breast cancer cases as compared to 11·5 % of the controls reported two or more deaths among such relatives. These variables were also examined by age (less than and more than 50 years old) and by family size; the same finding was obtained.

It could be assumed that the death of a family member residing in the same household as the subject might result in more of an emotional upset than the death of a non-household family member. Consequently, in Section B of Table VI the percentages are shown for cases and controls experiencing various numbers of deaths specifically among household members. There is essentially no difference: 12·8 % of the breast cancer cases experienced one or more deaths as compared to 11·7 % of the controls. The number of non-household deaths was also examined separately; again there was a remarkable similarity of cases and controls.

It is possible that death in different kinship categories would vary in the emotional effect on the subject. For example, the death of a husband or child might be expected to carry more traumatic impact than the death of a child's spouse, a sibling, or a more distant relative. Therefore, the relationship of the dead person to the respondent was examined in Section C of Table VI for the death which occurred closest in time to the date of diagnosis. Again no interesting differences were revealed. This table has to do with subjects 50 years of age and older. When subjects under 50 years of age were considered, the results were essentially the same.

Sections D and E of Table VI present information on the separations and divorces occurring in the families of the respondents. Section D shows that there was little difference in the proportion of cases and controls in whose families one or more separations or divorces had occurred during the five-year period prior to diagnosis. Section E examines the relationship of the divorced or separated relative to the subject. Although the numbers involved in this table are very small, there were no differences in either the proportion of cases and controls divorced or separated from their own spouses or the proportion of cases and controls having other relatives who were separated from their spouses.

Sections F, G, H, I, and J of Table VI provide information on illnesses in the families of subjects including: the total number of ill persons by age of subject; the number of ill persons in the subject's own household; the relationship of the ill person to the subject; whether the subject had to nurse the ill person and whether

TABLE VI.—*Insults Experienced by Breast Cancer Cases and Controls*

| Insults | Cases No. | Cases % | Controls No. | Controls % |
|---|---|---|---|---|
| A. Deaths among household and other family members | | | | |
| 0 | 208 | 59·1 | 395 | 59·3 |
| 1 | 103 | 29·3 | 195 | 29·2 |
| 2+ | 41 | 11·6 | 77 | 11·5 |
| Total | 352 | 100·0 | 667 | 100·0 |
| B. Deaths among household members only | | | | |
| 0 | 307 | 87·2 | 589 | 88·3 |
| 1+ | 45 | 12·8 | 78 | 11·7 |
| Total | 352 | 100·0 | 667 | 100·0 |
| C. Relationship of death closest in time to diagnosis of disease (Subjects 50 years and older) | | | | |
| Husband | 12 | 15·0 | 32 | 16·9 |
| Child | 2 | 2·5 | 7 | 3·7 |
| Parent | 12 | 15·0 | 19 | 10·1 |
| Sibling | 32 | 40·0 | 81 | 42·8 |
| Other | 22 | 27·5 | 50 | 26·5 |
| Total | 80 | 100·0 | 189 | 100·0 |
| D. Total number of separations and divorces | | | | |
| 0 | 336 | 95·5 | 612 | 91·8 |
| 1+ | 16 | 4·5 | 55 | 8·2 |
| Total | 352 | 100·0 | 667 | 100·0 |
| E. Relationship of divorced or separated relative | | | | |
| Own husband | 4 | 25·0 | 13 | 23·6 |
| Other relative's spouse | 12 | 75·0 | 42 | 76·4 |
| Total | 16 | 100·0 | 55 | 100·0 |
| F. Number ill in families (of subjects age 50 and over) | | | | |
| 0 | 57 | 26·3 | 92 | 19·5 |
| 1 | 49 | 22·6 | 106 | 22·5 |
| 2–3 | 69 | 31·7 | 151 | 31·9 |
| 4+ | 42 | 19·4 | 123 | 26·1 |
| Total | 217 | 100·0 | 472 | 100·0 |
| G. Number ill in subject's household (subjects age 50 and older) | | | | |
| 0 | 115 | 53·0 | 259 | 54·8 |
| 1 | 84 | 38·7 | 166 | 35·2 |
| 2–3 | 16 | 7·4 | 47 | 10·0 |
| 4+ | 2 | 0·9 | 0 | 0·0 |
| Total | 217 | 100·0 | 472 | 100·0 |
| H. Relationship of ill person to subject (illness closest in time to diagnosis) | | | | |
| Husband | 49 | 18·8 | 86 | 16·2 |
| Child, child's spouse | 27 | 10·4 | 105 | 19·7 |
| Parent or sibling | 120 | 46·2 | 233 | 43·8 |
| Other | 64 | 24·6 | 108 | 20·3 |
| Total | 260 | 100·0 | 532 | 100·0 |
| I. Did subject nurse the ill family member? | | | | |
| No | 198 | 76·7 | 420 | 81·6 |
| Yes, while working at job | 27 | 10·5 | 31 | 6·0 |
| Yes, held no outside job | 33 | 12·8 | 64 | 12·4 |
| Total | 258 | 100·0 | 515 | 100·0 |

TABLE VI.—*Continued*

| Insults | Cases No. | Cases % | Controls No. | Controls % |
|---|---|---|---|---|
| J. Number of illnesses subjects 50 years of age and older experienced | | | | |
| 0 | 103 | 47·5 | 244 | 51·5 |
| 1 | 71 | 32·7 | 147 | 31·0 |
| 2+ | 43 | 19·8 | 83 | 17·5 |
| Total | 217 | 100·0 | 474 | 100·0 |
| K. Number of relatives unemployed 1 month or longer | | | | |
| 0 | 279 | 79·5 | 473 | 71·3 |
| 1 | 41 | 11·7 | 119 | 17·9 |
| 2+ | 31 | 8·8 | 72 | 10·8 |
| Total | 351 | 100·0 | 664 | 100·0 |
| L. Relationship to subject of unemployed relative (unemployed closest in time to diagnosis) | | | | |
| Husband | 21 | 28·0 | 45 | 22·6 |
| Child, parent, sibling | 35 | 46·7 | 95 | 47·8 |
| Other | 19 | 25·3 | 59 | 29·6 |
| Total | 75 | 100·0 | 199 | 100·0 |
| M. Respondent's estimate of size of amount of money borrowed | | | | |
| None borrowed | 309 | 88·8 | 578 | 87·3 |
| Small amount | 19 | 5·5 | 34 | 5·1 |
| Large amount | 20 | 5·7 | 50 | 7·6 |
| Total | 348 | 100·0 | 662 | 100·0 |
| N. Subjects on welfare | | | | |
| No | 344 | 98·9 | 633 | 96·1 |
| Yes | 4 | 1·1 | 26 | 3·9 |
| Total | 348 | 100·0 | 659 | 100·0 |
| O. Periods family income perceived inadequate by subject | | | | |
| 0 | 256 | 73·6 | 450 | 67·9 |
| 1 | 91 | 26·1 | 209 | 31·5 |
| 2+ | 1 | 0·3 | 4 | 0·6 |
| Total | 348 | 100·0 | 663 | 100·0 |
| P. Number of places in which subjects have lived (subjects age 50 and over) | | | | |
| 1 | 155 | 72·1 | 353 | 75·6 |
| 2 | 45 | 20·9 | 64 | 13·7 |
| 3+ | 15 | 7·0 | 50 | 10·7 |
| Total | 215 | 100·0 | 467 | 100·0 |
| Q. Reason subject felt upset | | | | |
| No upset | 182 | 51·9 | 327 | 48·8 |
| Self ill | 26 | 7·4 | 42 | 6·3 |
| Others ill | 21 | 6·0 | 50 | 7·5 |
| Financial problems | 10 | 2·8 | 29 | 4·4 |
| Death in family | 24 | 6·8 | 35 | 5·3 |
| Insecurity feelings | 9 | 2·6 | 19 | 2·9 |
| Difficult relations with others | 37 | 10·5 | 65 | 9·8 |
| Any combination of above | 42 | 12·0 | 100 | 15·0 |
| Total | 351 | 100·0 | 667 | 100·0 |

this nursing took place at the same time the subject was employed outside the home; and the number of illnesses the respondent herself suffered.

Section F reveals that there was somewhat more illness in the families of the controls than the breast cancer cases. Thus, for subjects aged 50 and older, 80·5 % of the controls reported illness among relatives as compared to 73·7 % of the breast cancer cases. In addition, 26·1 % of the controls had four or more ill family members as compared to 19·4 % of the cases. This finding tends toward the opposite of what would be expected under the hypothesis of this study. When the same variable was examined for subjects under the age of 50 there was no difference between the cases and the controls.

Section G examines the number of persons ill in the subjects' own households for people 50 years and older. There was no difference between the cases and the controls concerning this variable. The same was true for younger subjects. Section H presents information on the relationship of the ill person to the subject for the illness which occurred closest in time to the diagnosis of the subject's present illness. The controls had more illness occurring among their children and the spouses of the children. Thus, 19·7 % of the controls had a child or child's spouse that was ill compared to 10·4 % of the breast cancer cases. No other interesting differences appeared.

Section I deals with nursing of the ill person by the subject and having nursed a relative while holding a job outside the household. Section J provides information on the number of illnesses the subject experienced. Neither table reveals any major differences between the cases and the controls.

Sections K, L, M, N, and O of Table VI present information on the economic stability of the families of cases and controls. Section K deals with the number of relatives in the respondents' families who were unemployed one month or longer. It reveals that 28·7 % of the controls had an unemployed relative compared to 20·5 % of the breast cancer cases, once again a finding opposite of what would be expected under the hypothesis. Little difference was found in the relationship of the unemployed relative to the subject, borrowing money and the size of the amount borrowed, and whether or not the subject was on welfare (see Sections L, M, and N). This was true regardless of age, size of family membership, and the location in time of the period of unemployment in relation to time of onset of symptoms of the present disease. Section O deals with the number of separate periods that family income was perceived as being inadequate by the respondent. There was a slight trend for the controls to have had more of such periods (32·1 % of the controls versus 26·4 % of the breast cancer cases).

Syme has found that geographic mobility is related to an increased incidence of coronary artery disease (Syme, Hyman and Enterline, 1965). We attempted to examine the impact of residential mobility by inquiring into the number of places in which subjects had lived in the five years prior to interview. As Section P of Table VI reveals, there was essentially no difference between the breast cancer cases and the controls in the proportion having lived in 1, 2, or 3 or more residences during the period under investigation. This was true regardless of age.

Some observers have suggested that the respondent's subjective assessment of whether or not he is upset may be more important in describing his status than the actual experience of traumatic events (Graham and Reeder, 1971). For this reason, we were interested in the extent to which the subjects, by their own report, felt upset, debilitated, and unduly tired for long periods.

No differences were found between cases and controls in the duration of feeling upset, the time of feeling upset as related to the onset of the symptoms of their present illness, and as Section Q shows, in the reasons expressed for feeling upset. We should point out that no attempt was made to determine whether the subjects were, by more reliable criteria, emotionally disturbed or upset. Our findings are based solely on the subjects' responses to the question, " Have there been periods when you felt upset? "

No difference was found in the number of hours of sleep of cases and controls either at night or in daytime naps. However, in examining the number of periods the subjects had felt extremely tired, by their own report, there was a difference. Somewhat more of the controls had experienced one or more tired periods compared to the breast cancer cases—once again a finding tending toward the opposite of

TABLE VIIA.—*Household and Non-Household Insults*

| | |
|---|---|
| Each household or non-household death | = 1 |
| Each household or non-household suicide | = 1 |
| Each household or non-household divorce or separation | = 1 |
| Each household or non-household illness (lasting 2 months or longer) | = 1 |
| Each household or non-household illness nursed by respondent while working | = 1 |
| Each period of household or non-household unemployment (lasting 3 months or longer) | = 1 |

TABLE VIIB.—*Respondent Insults*

| | |
|---|---|
| Each respondent illness | = 1 |
| Most recent illness—if it lasted two or more months | = 1 |
| Each experience of surgery by respondent | = 1 |
| If sleep regularly interrupted | = 1 |
| Each separate period respondent felt tired | = 1 |
| Each occupation held by respondent—over three | = 1 |
| If respondent did housework besides working | = 1 |
| Each period respondent felt upset for over two months | = 1 |
| Each period respondent felt family income inadequate | = 1 |
| Each time respondent borrowed money | = 1 |
| If respondent received home relief | = 1 |
| Each respondent membership in a religious organization (beyond 1) | = 1 |
| Each respondent membership in a non-religious organization | = 1 |
| Each different place respondent has lived—over 1 | = 1 |
| Each respondent marriage—over 1 | = 1 |
| Each respondent miscarriage | = 1 |

what would be expected under the hypothesis. There were no differences in the duration of such periods or in the time between experiencing such periods of fatigue and the onset of the subject's present illness.

In summary, a number of occurrences have been examined which could have been emotionally traumatic in the lives of a series of breast cancer cases and controls in the 5-year periods prior to the onset of symptoms of their disease. These occurrences included such objective life events as death and illness in the family, divorce and separation, economic problems, residential mobility, and more subjective incidents such as feelings of being upset or fatigued. In no case were interesting differences revealed.

*Cumulative insults experienced*

It could be asserted that although such individual instances of trauma might not singly be associated with pathology, combinations of such events could be so associated. For this reason, the cumulative numbers of various types of events

TABLE VIII.—*Number of Insults Experienced by Members of Subjects' Families Not Living in their Households*

| Number of insults | Cases No. | Cases % | Controls No. | Controls % |
|---|---|---|---|---|
| | Subjects < 50 years of age | | | |
| 0 | 68 | 50·4 | 93 | 47·5 |
| 1–2 | 46 | 34·1 | 64 | 32·6 |
| 3–4 | 14 | 10·4 | 21 | 11·2 |
| 5 or more | 7 | 5·1 | 17 | 8·7 |
| Total | 135 | 100·0 | 195 | 100·0 |
| | Subjects ⩾ 50 years of age | | | |
| 0 | 108 | 49·7 | 208 | 43·9 |
| 1–2 | 78 | 35·9 | 176 | 37·1 |
| 3–4 | 21 | 9·7 | 53 | 11·2 |
| 5 or more | 10 | 4·7 | 37 | 7·8 |
| Total | 217 | 100·0 | 474 | 100·0 |

TABLE IX.—*Number of Insults Experienced by Members of Households of Subjects*

| Number of insults | Cases No. | Cases % | Controls No. | Controls % |
|---|---|---|---|---|
| | Subjects < 50 years of age | | | |
| 0 | 86 | 63·7 | 125 | 63·8 |
| 1–2 | 39 | 28·9 | 53 | 27·0 |
| 3–4 | 9 | 6·7 | 14 | 7·2 |
| 5 or more | 1 | 0·7 | 4 | 2·0 |
| Total | 135 | 100·0 | 196 | 100·0 |
| | Subjects ⩾ 50 years of age | | | |
| 0 | 137 | 63·1 | 293 | 61·8 |
| 1–2 | 64 | 29·5 | 147 | 31·0 |
| 3–4 | 13 | 6·0 | 29 | 6·1 |
| 5 or more | 3 | 1·4 | 5 | 1·1 |
| Total | 217 | 100·0 | 474 | 100·0 |

TABLE X.—*Number of Insults Experienced by Subjects*

| Number of insults | Cases No. | Cases % | Controls No. | Controls % |
|---|---|---|---|---|
| | Subjects < 50 years of age | | | |
| 0–1 | 10 | 7·4 | 10 | 5·1 |
| 2–5 | 56 | 41·5 | 72 | 36·8 |
| 6–9 | 49 | 36·3 | 78 | 39·7 |
| 10–13 | 15 | 11·1 | 28 | 14·3 |
| 14 or more | 5 | 3·7 | 8 | 4·1 |
| Total | 135 | 100·0 | 196 | 100·0 |
| | Subjects ⩾ 50 years of age | | | |
| 0–1 | 8 | 3·7 | 19 | 4·0 |
| 2–5 | 96 | 44·1 | 215 | 45·3 |
| 6–9 | 82 | 37·8 | 164 | 34·6 |
| 10–13 | 30 | 13·9 | 65 | 13·7 |
| 14 or more | 1 | 0·5 | 11 | 2·4 |
| Total | 217 | 100·0 | 474 | 100·0 |

experienced by members of subjects' families and by themselves were examined. Tables VIIA and VIIB show the specific insults examined in Tables VIII–XI. Table VIII presents the proportion of cases and controls less than 50 years of age and 50 years of age and older experiencing various numbers of insults among non household family members. For the 50 years and older age groups, there is a slight tendency for the controls to have experienced more insults than the breast cancer cases. Thus, 56·1 % of the controls experienced one or more insults as compared to 50·3 % of the cases. Table IX shows the percentage of cases and controls, by age, experiencing various numbers of insults among household family members. Essentially no differences appear.

Table X considers the total number of insults occurring to subjects themselves, including such potential traumas already discussed as divorce or death of their own

TABLE XI.—*Total Number of Insults Experienced by Subjects and their Household and Non-Household Family Members*

| Number of insults | Cases No. | Cases % | Controls No. | Controls % |
|---|---|---|---|---|
| | Subjects < 50 years of age | | | |
| 0–2 | 11 | 8·1 | 13 | 6·6 |
| 3–6 | 51 | 37·9 | 56 | 28·5 |
| 7–10 | 41 | 30·4 | 65 | 33·3 |
| 11–14 | 20 | 14·8 | 40 | 20·4 |
| 15–18 | 10 | 7·4 | 16 | 8·2 |
| 19–24 | 1 | 0·7 | 6 | 3·0 |
| 25 or more | 1 | 0·7 | 0 | 0·0 |
| Total | 135 | 100·0 | 196 | 100·0 |
| | Subjects ⩾ 50 years of age | | | |
| 0–2 | 10 | 4·6 | 29 | 6·1 |
| 3–6 | 78 | 35·9 | 164 | 34·6 |
| 7–10 | 80 | 36·8 | 163 | 34·4 |
| 11–14 | 33 | 15·2 | 74 | 15·6 |
| 15–18 | 14 | 6·5 | 34 | 7·2 |
| 19–24 | 2 | 1·0 | 10 | 2·1 |
| 25 or more | 0 | 0·0 | 0 | 0·0 |
| Total | 217 | 100·0 | 474 | 100·0 |

spouse, periods of feeling tired and upset, and of being ill themselves. Also included in this table were other events which could be interpreted as traumatic by the individual. These consisted of: (a) customarily having sleep interrupted; (b) high occupational mobility, interpreted as having had more than three jobs in the five years prior to symptom onset; (c) working outside the house and also being responsible for housework; and (d) having a number of religious and other organizational memberships, in addition to a single affiliation with a church or temple.

For subjects under age 50 there is once again a tendency for the controls to have experienced more of such insults. Thus, 58·1 % of the controls experienced six or more insults compared to 51·1 % of the cases. There are no differences for the subjects 50 years and older. Table XI considers the total number of insults occurring to subjects themselves and to household and non-household family members. For the subjects under age 50, there seemed to be a tendency for the control series to include a slightly larger proportion of women who had experienced greater numbers of insults.

It is likely that traumatic events occurring to oneself or to members of one's own household may have more emotional impact than those happening to more distant relatives. For this reason, we conducted the analysis exhibited in Table XII. This table presents data for cases and controls, by age, in terms of numbers of insults experienced, weighted for the closeness of the relationship of the individual experiencing the insult to the respondent. The number of incidents occurring to the respondent herself was weighted four times as heavily as those occurring to family members not living in her household; and those occurring to household members were weighted twice as heavily as those occurring to family members not residing in her household. Again, no particularly large differences are observed between cases and controls.

TABLE XII.—*Total Number of Insults Experienced, Weighted* for Closeness of Relationship to Subjects*

| Weighted number of insults | Cases No. | Cases % | Controls No. | Controls % |
|---|---|---|---|---|
| | Subjects $<50$ years of age | | | |
| 1–5 | 5 | 3·7 | 5 | 2·6 |
| 6–20 | 43 | 31·9 | 60 | 30·6 |
| 21–40 | 62 | 45·9 | 91 | 46·4 |
| 41–55 | 20 | 14·8 | 26 | 13·3 |
| 56 or more | 5 | 3·7 | 14 | 7·1 |
| Total | 135 | 100·0 | 196 | 100·0 |
| | Subjects $\geqslant 50$ years of age | | | |
| 0–5 | 5 | 2·3 | 14 | 3·0 |
| 6–20 | 76 | 35·0 | 163 | 34·4 |
| 21–40 | 105 | 48·4 | 218 | 45·9 |
| 41–55 | 23 | 10·6 | 60 | 12·7 |
| 56 or more | 8 | 3·7 | 19 | 4·0 |
| Total | 217 | 100·0 | 474 | 100·0 |

* Weighting scheme used:
Total number of insults experienced by household members = a
Total number of insults experienced by non-household members = b
Total number of insults experienced by the respondent = c
Weighted total insults = $2\Sigma a + \Sigma b + 4\Sigma c$.

### DISCUSSION

It would thus appear that there is no significant difference between breast cancer cases and controls in the experiencing of single or cumulative numbers of insults by either members of their families or by themselves. The similarity between cases and controls is indeed remarkable. We were moved to undertake this investigation by suggestions from two lines of inquiry, psychophysiology and epidemiology. The first speculates that breast cancer may be related to a variety of abnormal psychological states, ranging from early relationships with parents to having experienced misfortune subsequently. Small numbers, infrequent use of controls, little replication employing good research design, lack of definitions of entities studied, and vagueness in description of methods of measurement characterize reports of these studies. These faults, however, should not obscure the likelihood that the body does respond to emotional states engendered by the social situation. This response partly may be endocrine.

Epidemiologists have indicated an endocrine aspect to the etiology of breast cancer. The declining rate of increase after menopause, the lowered risk associated with artificial menopause, reduced menstrual function, and early pregnancy found in a variety of studies are examples. These suggested the possibility of an increased risk accompanying the experiencing of social trauma which could induce endocrinological effect. It is significant, then, that there was no increased risk discovered in this series: cases and controls could hardly have been more similar in their having experienced deaths in the family, unemployment, and the other social trauma investigated.

Nevertheless, our investigation is open to several criticisms. First, we are relying on patients' own retrospective reports concerning the occurrence of incidents in their families such as death, illness, and the like. It is quite possible that there could be under-reporting of such incidents. There is no evidence, however, that the under-reporting occurs more frequently among either the cancer or the control patients.

To avoid the subjectivity inherent in relying upon patients' assessments of their own emotional status, we queried regarding specific, concrete, easily remembered events, such as deaths, divorces, borrowing money, unemployment, and residential mobility. But in avoiding the subjectivity of some research designs, we have only cursorily examined the meaning these events had for the subjects. It may be argued that these events may mean different things to different people. On the other hand, in most cases the events considered here are likely to be interpreted as traumatic emotionally.

Again, in our inquiry regarding subjects experiencing various numbers of events, we have simply counted numbers in some analyses (Tables VIII–XI) and used a weighting scheme (Table XII) which, while it attempts to account for closeness of occurrence of the event to the subject, nevertheless is arbitrary. Regardless, most subjects would probably agree that untoward events happening to oneself or one's immediate family are greater in emotional impact than those occurring in the extended family.

Although our observations included rather substantial numbers of cases and controls, it must be emphasized that we compared breast cancer patients with individuals with non-neoplastic illnesses who were hospitalized in Roswell Park Memorial Institute. We are thus comparing persons ill with one disease to persons ill with other diseases. But if one hypothesizes that social trauma precedes the onset not only of breast cancer but of a wide variety of diseases, one would expect the finding we obtained. We find it difficult to believe, however, that so many different conditions would share the same etiological factors. Nevertheless, a more valid comparison would have been between patients with cancer of the breast from all hospitals in the community and healthy controls from the community.

There are, thus, several circumstances that could invalidate our findings. The lack of relationship which we discovered is unique among studies published to date. On the other hand, so are the methods: we have used larger numbers of cases and controls than previous studies, have queried regarding concrete, easily remembered events, and have attempted to measure relationships with multiple exposures to such events. We hope that future research would repair some of our deficiencies, particularly in exploring the meaning of events to subjects and in using samples of the universe of well people in a community to serve as controls for cases from all hospitals in the community.

### REFERENCES

Bahnson, C. B.—(1969) *Ann. N.Y. Acad. Sci.*, **164**, 319.
Graham, S., Levin, M. and Lilienfeld, A. M.—(1960) *Cancer, N.Y.*, **13**, 180.
Graham, S. and Reeder, L. G.—(1971) Social Factors in Chronic Illnesses, Chapter 3 in ' Handbook of Medical Sociology ' edited by Freeman, H. E., Reeder, L. G. and Levine, S. New York (Prentice-Hall, Inc.).
Levin, M. L., Sheehe, P. R., Graham, S. and Glidewell, O.—(1964) *Am. J. publ. Hlth*, **54**, 580.
Lilienfeld, A. M.—(1956) *Cancer, N.Y.*, **9**, 927.—(1958) *J. chron., Dis.*, **8**, 649.
Lilienfeld, A. M. and Johnson, E. A.—(1955) *Cancer, N.Y.*, **8**, 875.
MacMahon, B. and Feinleib, M.—(1960) *J. natn. Cancer Inst.*, **24**, 733.
Salber, E. J., Trichopoulos, D. and MacMahon, B.—(1969) *J. natn. Cancer Inst.*, **43**, 1013.
Shapiro, S., Strax, D., Venet, L. and Fink, R.—(1968) *Am. J. publ. Hlth*, **58**, 820.
Syne, S. L., Hyman, M. M. and Enterline, P. E.—(1965) *J. Hlth hum. Behav.*, **6**, 178.
Tarlau, M. and Smalheiser, I.—(1951) *Psychosom. Med.*, **13**, 117.
Wheeler, J. I. and Caldwell, B. M.—(1955) *Psychosom. Med.*, **17**, 256.